# Supplementary material for: Dynamically tuning friction at the graphene interface using the field effect
Source: Nat Commun. 2023 Sep 19;14:5801. doi: 10.1038/s41467-023-41375-7 (PMC10509204; doi:10.1038/s41467-023-41375-7)
Supplement: Supplementary file 1 — Supplementary Information [file 41467_2023_41375_MOESM1_ESM.pdf]

Dynamically Tuning Friction at the Graphene Interface Using the Field Effect

Gus Greenwood,[1] Jin Myung Kim,[2,3] Shahriar Muhammad Nahid,[4] Yeageun Lee,[4] Amin Hajarian,[3] SungWoo Nam,[3] Rosa M. Espinosa-Marzal[1,2*]
[1]Department of Civil and Environmental Eng. University of Illinois at Urbana-Champaign, Urbana, IL, 61801, USA
[2]Department of Materials Science and Eng. University of Illinois at Urbana-Champaign, Urbana, IL, 61801, USA
[3]Department of Mechanical and Aerospace Engineering, University of California, Irvine, Irvine, CA, 92697, USA
[4]Department of Mechanical Science and Eng. University of Illinois at Urbana-Champaign, Urbana, IL, 61801, USA
*Corresponding author, e-mail: rosae@illinois.edu
These authors contributed equally: Gus Greenwood, Jin Myung Kim

Supplementary Information

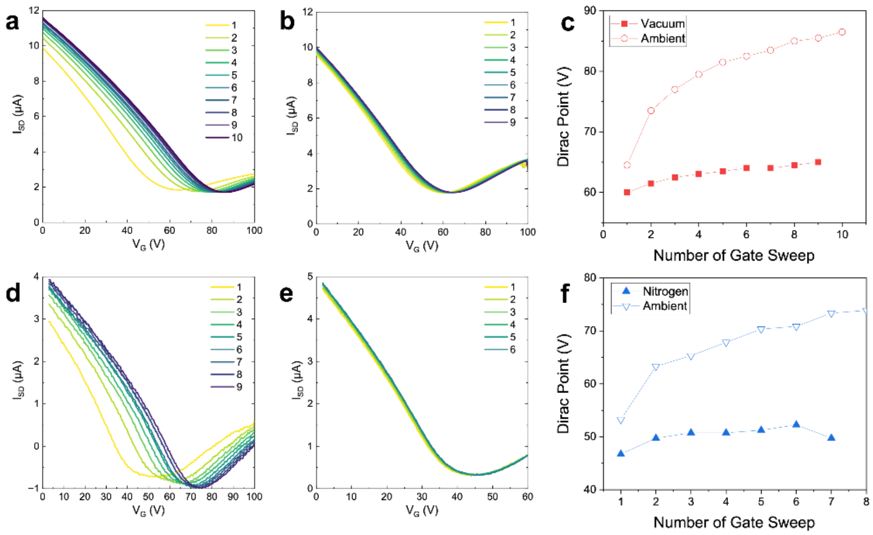

Supplementary Figure 1. Transfer characteristics of graphene FET measured in different environments. **a-c** Charge transport measurements for graphene FET in (**a**) ambient condition and (**b**) vacuum (10^{-5} Torr), and (**c**) summary of shift of Dirac point as a function of number of gate sweeps. **d-f** Transfer characteristics of a different graphene FET sample, measured in (**d**) ambient condition and (**e**) nitrogen purging atmosphere, and (**f**) the plot showing Dirac point shift. For all measurements, source-drain bias was kept constant at 50 mV.

Effects of ambient condition on the Dirac point of graphene FETs:

Application of strong electric field to graphene in ambient condition causes irreversible physisorption of oxygen and/or water molecules. These impurities inevitably dope the graphene sheet resulting in a shift in the Dirac point. This is evident from Figure S1a. Under repeated sweeping of gate bias from 0 V to 100 V, the Dirac point shifts from an initial value of 64 V to 85 V. This shift of Dirac point is permanent and does not recover after eliminating the gate bias, indicating an irreversible n-doping of graphene channel. However, the physisorption of ambient molecules is avoided if the transport measurements are performed in high vacuum or continuous purging with N_2. As shown in Supplementary Figure 1b and c, the Dirac point shift is below 5 V in vacuum. N_2 atmosphere also prevents the Dirac point shift, as illustrated in Figures S1e and f. All our scanning probe measurements of friction, adhesion, and electrostatic interaction were performed under continuous purging with dry N_2 where the Dirac point remains stable, even with successive sweeping of gate bias.

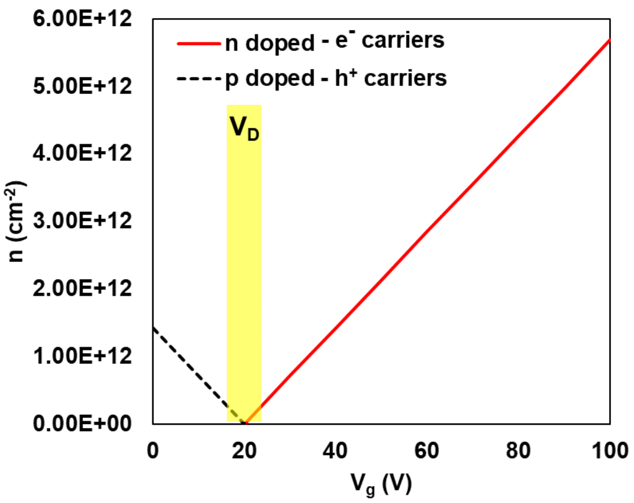

Supplementary Figure 2. Calculated charge carrier density (n) for the graphene FET device described in Figure 1 of the main text, assuming a Dirac point of 20 V. This estimation only accounts for the gate induced carrier density because thermally induced carriers are calculated to have induced densities ~2 orders of magnitude smaller at experimental temperatures (25° C). Moving from one side of the Dirac point to the other changes the charge carrier identity from electrons to holes, as noted in the figure legend. Devices with different Dirac points will shift the location of minimum charge density $n = 0$ along the x-axis. Note that very near the Dirac point the charge density relationship becomes nonlinear as thermally induced carriers become more important but this detail is not accounted for in the plot.

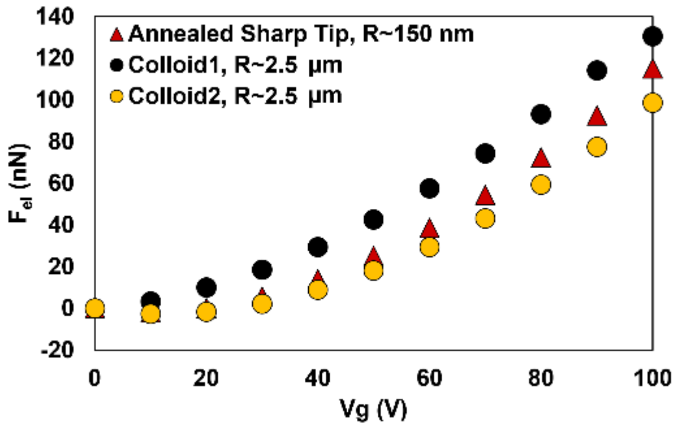

Supplementary Figure 3. Electrostatic interaction between graphene and insulating tips. The magnitude of F_{el} is determined using the difference between the deflection of the tip at 0 V (baseline uninfluenced by electrostatics) and a given gate voltage both at 3.5 μm from the surface (see Methods and Supplementary Figure 13). The data are taken from the experiments used in Figure 2 for insulating tips, corresponding to experiments I1, I2, and I3. For simplicity of this comparison only the increasing voltage sweep is shown. The plot compares results taken with a thermally annealed sharp tip with a radius of 150 nm (red triangles) and two colloids with radii of 2.5 μm (black and yellow circles). The electrostatic interaction measured at 3.5 μm from the surface is negligibly influenced by the type and size of the insulating tips. As also shown in Figure 2d;g, the friction force between graphene and the insulating tips is qualitatively similar when measured with a colloid and a thermally annealed tip. The contact radius between graphene and a silica colloid with a radius of 2.5 μm at 10 nN applied normal load is 5.4 nm, whereas it is 2.12 nm for a tip with a radius of 150 nm (a factor of 2 smaller), and hence, one would expect higher friction with the colloid (experiments I1 and I2), compared to the sharp tip (I3). However, asperities on the colloid surface are very common and may lead to smaller contact radii that would justify the results. Based on these results, we conclude that the difference in size among the insulating tips is not a major influencing factor on our experimental data. It does influence the magnitude of friction and adhesion, but the results are qualitatively similar.

Electrostatic interaction at contact

The electrostatic force increases with decreasing distance to the surface.[1] Force-distance curves collected at the range of gate voltages reveal that, for insulating tips, this method underestimates the true electrostatic attraction at contact (the force at 0 tip-surface separation) by a factor of ~3, implying that the insulating tip eCoF displayed in Figure 4 is *over*estimated. For the Si tips, the electrostatic attraction at contact is only a factor of ~1.1 larger than that measured 3.5 μm from the surface. This supports that even though the electrostatic attraction at contact (a more directly relevant value for friction measurements) is not measured *in situ* for every condition discussed here, the conclusions related to the eCoF and friction tunability with Si tips still hold true.

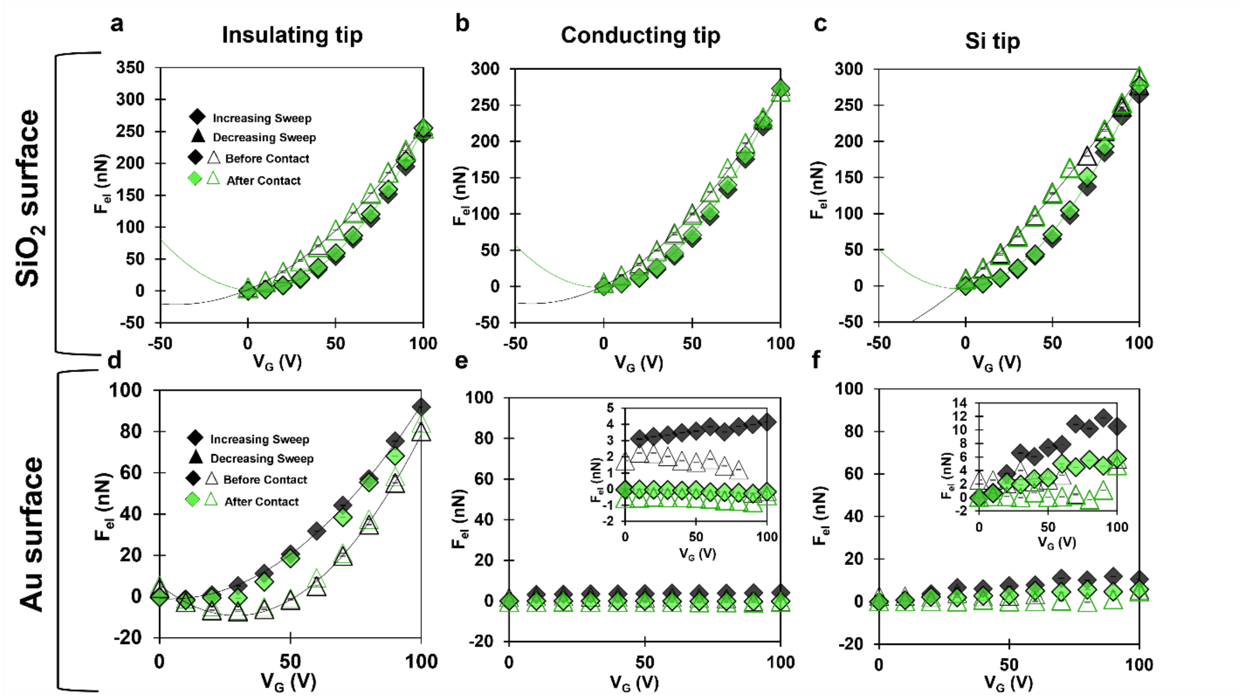

Supplementary Figure 4. Electrostatic interaction between AFM tip and reference surfaces. **a-c**SiO_2 surfaces with **(a)** insulating (a silica colloid tip for both surfaces), **(b)** conducting and **(c)** Si tips. **d-f** Gold-coated surfaces with **(d)** insulating, **(e)** conducting and **(f)** Si tips. The insets in **(e)** and **(f)** show the same data with a different Y axis. Error bars in all panels are the standard deviation of ~5 seconds of steady tip deflection collected at 10 Hz at fixed backgate voltage and distance from surface.

Electrostatic interaction between reference surfaces and AFM tips

The strongest interaction is measured between SiO_2 surfaces and all three types of tips. In all cases, the non-contact force changes parabolically with backgate potential, as expected for an electrostatic interaction (Supplementary Figure 4a-c). The origin of this force is due to the polarization of the SiO_2 surface by the electric field. The minimum of the parabola shifts to the left during decreasing potential sweeps, which is attributed to trapped charges. The tip size/type does not influence the non-contact electrostatic interaction between tip and the reference SiO_2 surface. The behavior is similar to that obtained for graphene with insulating tips, but the electrostatic force is always weaker in the latter case (~1/3). The attraction between insulating tips and reference gold-coated (conducting) surfaces stays parabolic but is reduced, as in the case of graphene (Figure 2b). The electrostatic interaction between a reference conducting surface and conducting tips significantly deviates from the results on graphene, as the attraction is very small and the parabolic dependence is lost (Supplementary Figure 4e), similar to the results with Si tips (Supplementary Figure 4f). Such behavior is attributed to the reduction of the electric field between gold and tip due to charge transfer. This is, indeed, similar to the interaction measured between conducting tips and graphene (Figure 2b).

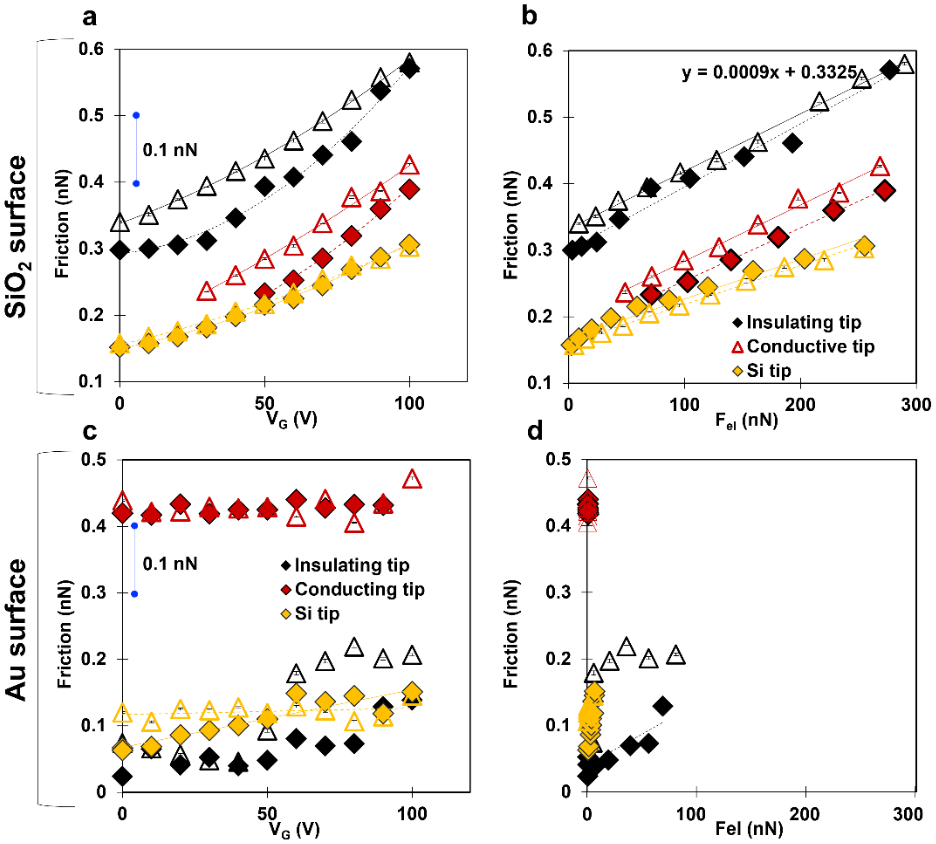

Supplementary Figure 5. Friction as a function of V_G and of F_{el} for reference surfaces. a-b Results with SiO_2 surfaces and three types of tips. Lines in A and B are examples of parabolic and linear fits, respectively. **c-d** Results with gold surfaces and three types of tips. The legend in B applies to all plots. **a, c** Friction as a function of the applied backgate potential. **b, d** Friction as a function of the electrostatic attraction measured after friction measurements. Diamonds indicate the increasing sweep and triangles the decreasing sweep for each tip type. Error bars, usually small and within the symbols, indicate the standard deviation of 6 repeated friction line scans in the same location.

Measurements on SiO_2 surfaces with Si and conducting tips show the same trends as with insulating tips (Supplementary Figure 5a-b): $F_{el} \propto V_G^2$, $F \propto V_G^2$, and $F \propto F_{el}$. This confirms that the polarization of the insulating surface by the electric field dictates the interaction between tip and surface, independently of the conducting characteristics of the tip. When the Si tip slides on a conductive surface (Au-coated silicon wafers), the tunability of friction via the electric field is lost (Supplementary Figure 5c-d). The clustering of data points as a function of the electrostatic interaction –as also found on graphene with conductive tips – lets us conclude that the major underlying mechanism is the screening of the electric field and reduction of the electrostatic interaction, as also found on graphene with conductive tips.

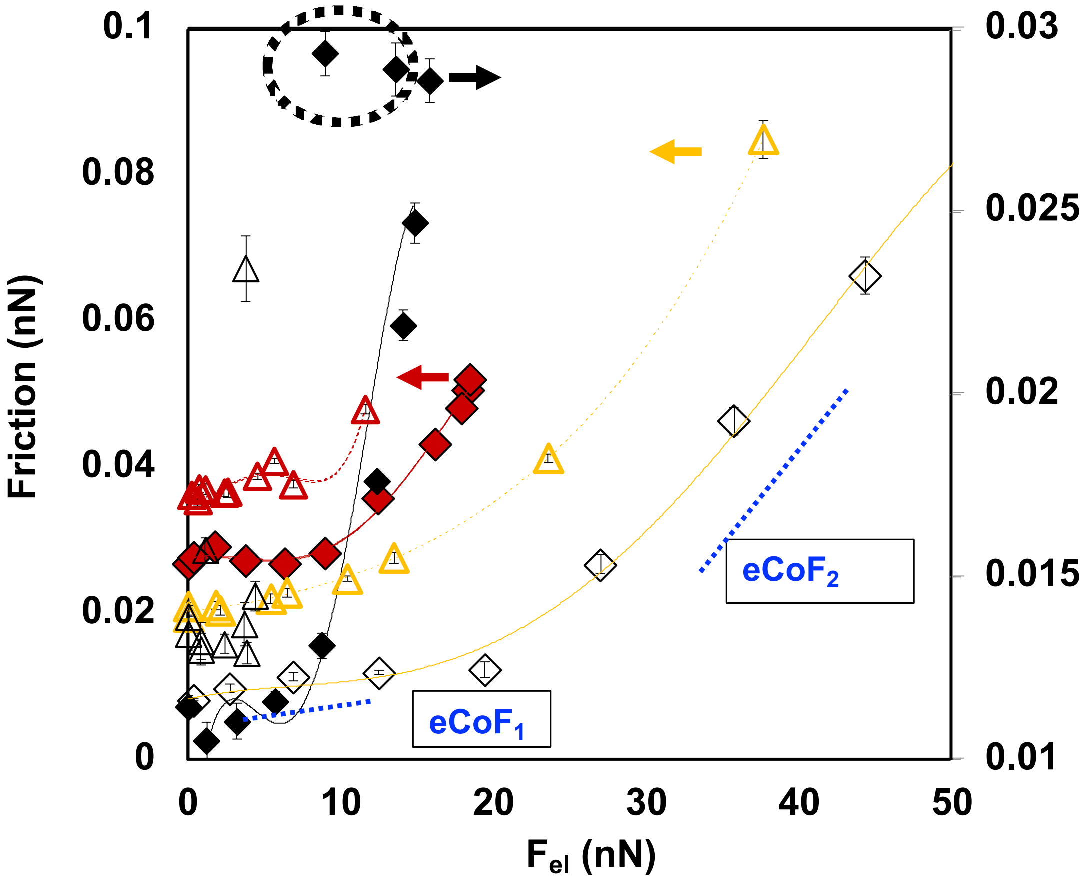

Supplementary Figure 6. Friction between graphene and Si tips as a function of the electrostatic attraction measured before friction measurement. Figure 2I shows a clustering before the potential is reversed, when using the electrostatic force "after" friction measurements. This clustering vanishes when the friction force before contact is used, which indicates that the clustering is directly related to the charge transfer in contact. Diamonds indicate the increasing sweep and triangles the decreasing sweep for each measurement, with each color representing a different tip-sample combination. Error bars, often small and within the symbols, indicate the standard deviation of 6 repeated friction line scans in the same location.

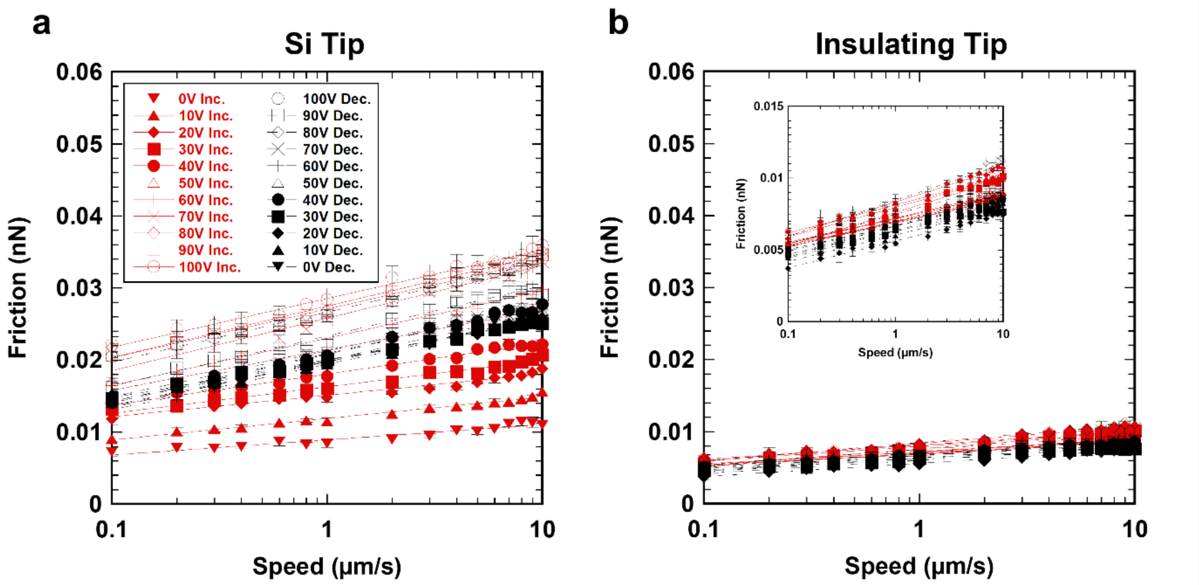

Supplementary Figure 7. Friction force measured on graphene vs. velocity as a function of the backgate potential during increasing (red) and decreasing sweeps (black). **a** Friction measured using a Si tip (R ~ 55 nm) **b** Friction measured using an insulating tip (R ~ 150 nm) The inset of (**b**) shows the same data with different y-axis scale. Load applied= 10 nN. Error bars, often small and within the symbols, indicate the standard deviation of 6 repeated friction line scans in the same location.

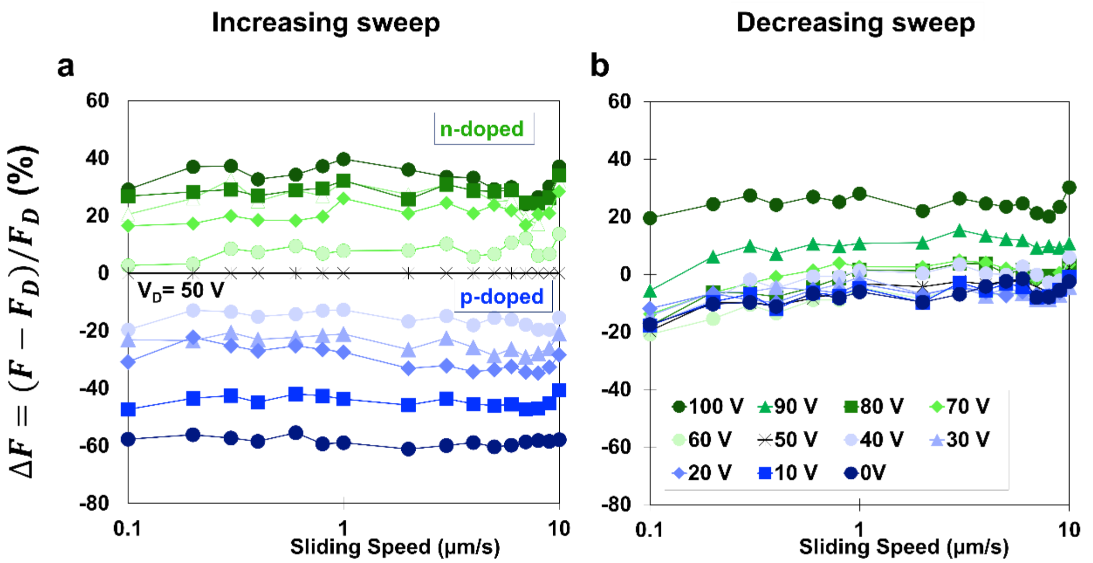

Supplementary Figure 8. Excess friction compared to the Dirac point for a Si tip sliding on graphene. **a** Increasing voltage sweep. **b** Decreasing voltage sweep. Color schemes match the main text, with n-doped graphene in shades of green and p-doped in shades of blue. Error bars, usually smaller than the symbols, indicate the standard deviation of 6 repeated friction line scans in the same location propagated through the calculation of ΔF.

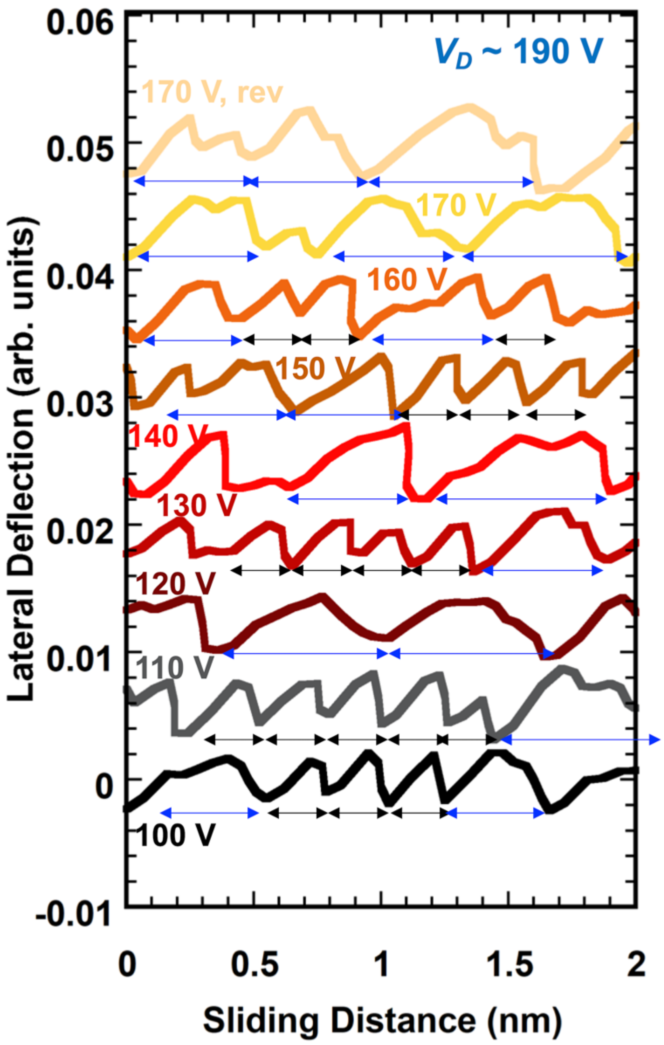

Supplementary Figure 9. Example of stick slip as a function of potential below the Dirac point (~190-200 V for this sample) on a graphene/hBN/SiO_2 FET device. Measurements were only performed at voltages below the Dirac point here to avoid breakage of the dielectric at high gate voltages. The high Dirac point of this sample is higher compared to that of samples used for the main friction results due to ambient contaminants and the higher difficulty of fabricating the hBN samples. Black arrows indicate single slip events while blue indicate double and triple slips. In this case, without crossing the Dirac point, there is no change in the average height of the stick slip pattern, in contrast to when the Dirac point is crossed in Figure 6a of the main text.

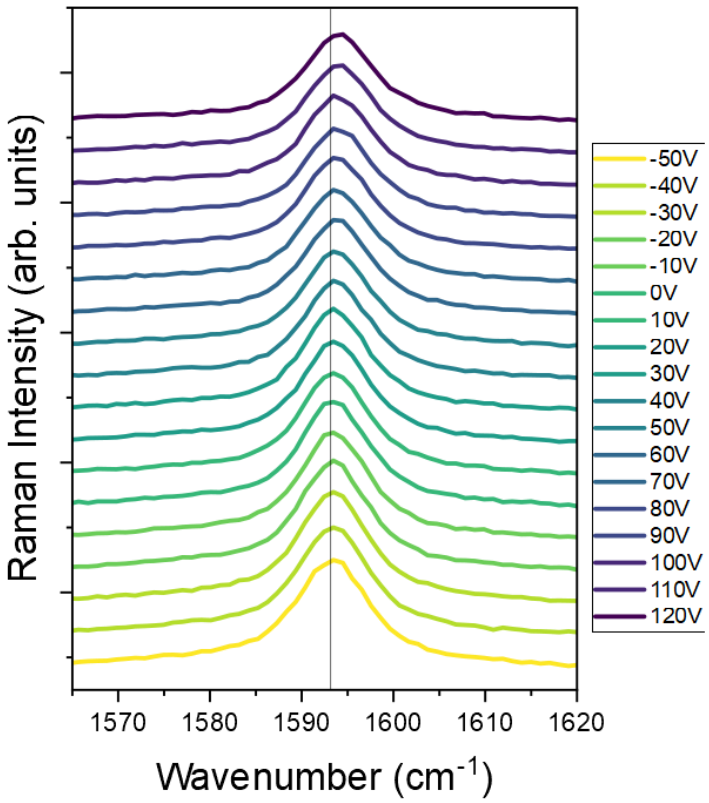

Supplementary Figure 10. Raman spectroscopy as a function of the gate bias for a graphene microchannel FET device with a Dirac point at 20 V. A 532 nm continuous-wave laser (P = 1 mW) was focused on a microchannel graphene FET (L = 20 µm, W = 30 µm) for 30 seconds under constant gate bias. We swept gate bias from –50 V to 120 V for each spectrum.

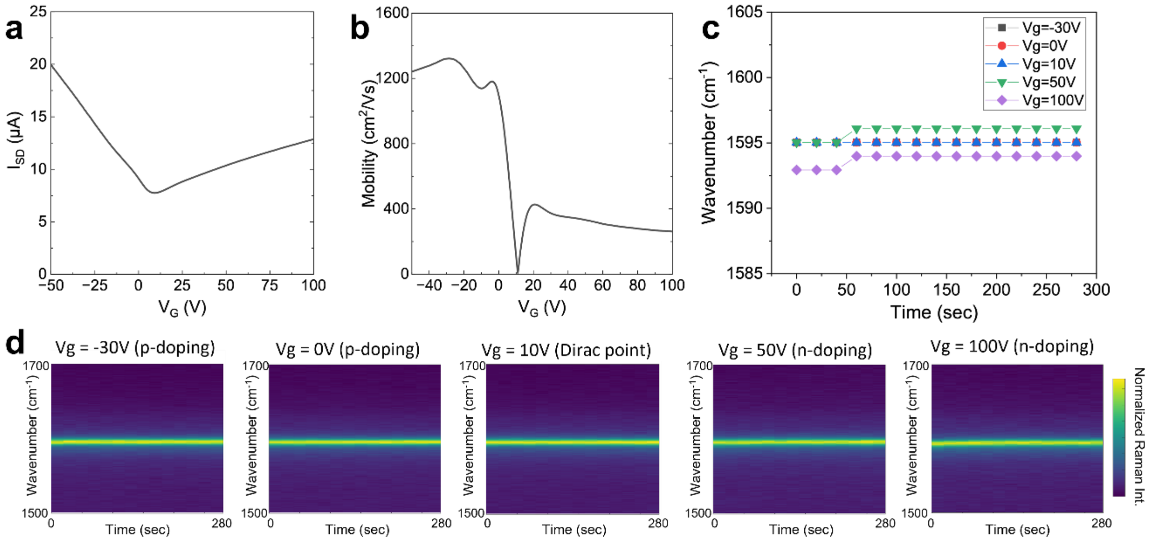

Supplementary Figure 11. Time-dependent Raman spectroscopy measurements. **a** Transfer characteristics of microchannel graphene FET in ambient environment with continuous forward/reverse sweeping of gate bias (with V_{SD} = 50 mV). **b** Field-effect mobility of graphene FETs. **c** Time-dependent shift of the G peak over time as a function of the back gate potential. At back gate potentials of 50 and 100 V, there is a peak shift after ~50 seconds, which is attributed to trapped charges. **d** Time-dependent Raman spectra. The maps show the G peak (position and intensity) as a function of time and back gate potential. Source-drain bias (V_{SD}) was maintained at 1 mV.

Potential-and time dependent Raman spectroscopy

Both phononic and electronic contributions to friction have been also shown to be temperature dependent.[2] To exclude the influence of thermal effects, time-dependent Raman spectroscopy with a 532 nm CW laser (power = 1 mW) and 1800 lpmm (lines per mm) grating spectrometer (XperRAM, Nanobase, South Korea) was performed on microchannel graphene field-effect transistors that were fabricated via photolithography (L = 100 µm, W = 30 µm) and exhibited a Dirac point at 10 V (Supplementary Figure 11a,b). The sample was prepared with commercial graphene (Grolltex) and thermally annealed at 450 ºC. The same fabrication was used for the samples used for the measurements displayed in Supplementary Figure 10 and Figure 6b. The Raman signal was collected at a fixed position every 20 seconds until the total accumulated time reached 5 minutes. The gate bias was varied from -30 V (p-doping) to 100 V (n-doping), and we kept the gate bias constant for each set of measurements. The measurements were carried out in ambient environment.

Because the G peak exhibits a temperature-sensitive shift of -0.016 cm^{-1}/K,[3] the heating of the sample surface should be reflected in a gradual negative shift of the G peak. Supplementary Figure 11c displays the G peak position and its intensity as a function of measurement time,

while the G peak shifts are summarized in Supplementary Figure 11d. The results show no correlation between the G peak position and the time under gate bias. We only observed an abrupt positive shift in the case of n-type doping (V_G = 50 V, 100 V), but the peak became saturated in less than 1 min. This shift is attributed to the increase in surface charge traps under ambient environment.[4] It is possible that the heating leads to a small negative shift (<-0.3 cm^{-1}) that is not detected with the precision of the spectrometer, implying a temperature increase <20 K. Because the influence of the temperature on atomic scale friction is sublinear,[5] this could not explain the significant increase in the friction force above the Dirac point. Although hot-carrier induced heating of graphene devices has been reported earlier, this occurred by Joule heating under high source-drain bias and direct current flow.[6,7] For instance, a source-drain bias of 1 V can increase the temperature of a graphene FET device by 100 K, with maintaining a linear relationship between source-drain bias and temperature increase up to 1000 K.[8] Our friction measurements were performed under low source-drain bias (V_{SD}=1 mV). Although we applied high gate bias, the power in the graphene channel is limited to several nW, which can exclude heating. We also note that, if there were less than 100 K temperature increase, it would not affect the doping level or carrier density in graphene FET, since doping-induced carrier greatly exceeds the thermally induced graphene at room temperature. Thus, we conclude that the gate bias does not have a significant effect on sample heating during *in situ* friction measurements.

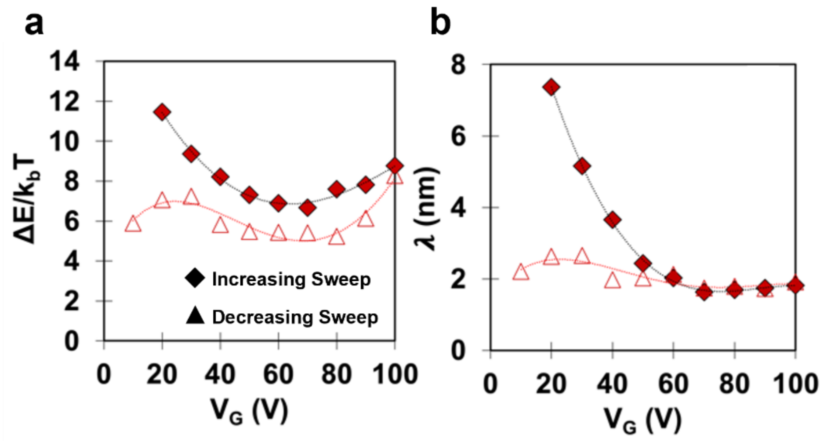

Supplementary Figure 12. Model parameters to describe friction excess $\Delta F'$ via the Bell and Eyring model. a energy barrier ΔE and **b** shear activation length λ. The model parameters at 10 V during forward sweeping are not shown because Eq. (1) did not provide a good fit to the experimental data.

Analogy to the viscous dissipation originating from hydrodynamic electron flow:
The Bell and Eyring model predicts a logarithmic dependence of friction on velocity, which we apply here to $\Delta F'$ per analogy:

$$\Delta F' = \frac{E_a}{\lambda} + \frac{k_B T}{\lambda} \ln\left(\frac{V}{V_0}\right) = \frac{\Delta E}{\lambda} + \frac{k_B T}{\lambda} \ln V \tag{1}$$

where V is the sliding velocity of the tip, E_a is the energy barrier for the tip to slide from one energy minimum to the next, λ is the shear activation length, k_B is the Boltzmann constant, and T is the temperature of the system (in this case 298 K) and V_0 a reference velocity.

This logarithmic relation is attributed to a shear-promoted thermally activated slip in the context of transition state theory. [8] This theory was applied by Eyring to describe liquid viscosity at the molecular level based on activated flow and is often applied to describe the origin of friction in thin-film lubrication. That is, for slip to occur, the liquid molecule, initially in an equilibrium position (an energy minimum) needs to jump over a "transition state" before reaching the adjacent energetic minimum, which requires an activation energy. Although the thermal energy of the molecules might be sufficient to overcome this energy barrier, the shear force applied by the tip on the molecule reduces the energy barrier, and thereby, promotes the slip; when the molecule falls in the adjacent minimum, the applied work is irreversibly dissipated (lost).

There are a couple of important caveats to this model. First, Eyring's model is traditionally used in the context of liquid viscosity. We invoke it here as analogous by considering this model accounts for the energy dissipation due to the carrier flow against the tip. there is no evidence that the dissipation is analogous. Second, the potential energy surface envisioned is usually due to the atomic lattice of the surface, with the AFM tip functioning as a single asperity sliding

between lattice points. Due to the larger size of the used AFM tips, we assume that the parameters extracted from Eq. 1 are averages over multiple minima. Because of this, λ ends up taking values in the range of nanometers as opposed to Angstroms. Third, the reference velocity V_0 is unknown, and hence, we cannot determine the energy barrier but only $\Delta E = E_a - k_B T \ln V_0$. We assume that V_0 is the same for all values of V_G, and compare the values of ΔE.

Note that Eq. (1) does not describe well $\Delta F'$ for contacts between graphene and insulating tips, which is consistent with our previous results: the friction force measured with insulating tips is not sensitive to the carrier density or doping state of graphene, and hence, the presumed viscous dissipation is not relevant in this case. In contrast, Eq. (1) fits the experimentally determined excess friction $\Delta F'$ for Si tips sliding on graphene very well. The parameter λ as function of V_G decreases from ~7 to 2 nm during the increasing sweeps, while it varies only slightly ~1.7-2.6 nm during the decreasing sweep, reflecting the trapped state of the sliding interface (Supplementary Figure 12a). The value of ΔE changes non-monotonically with V_G with a minimum at ~70V, close to the macroscopic Dirac point of 50-60 V (Supplementary Figure 12b). The lines in the plot are a polynomial function of order 4 to guide the eyes. These results suggest that the electric-field effect alters both the energy barrier for sliding and the shear-activation length at the interface between graphene and the Si tip, and thereby friction.

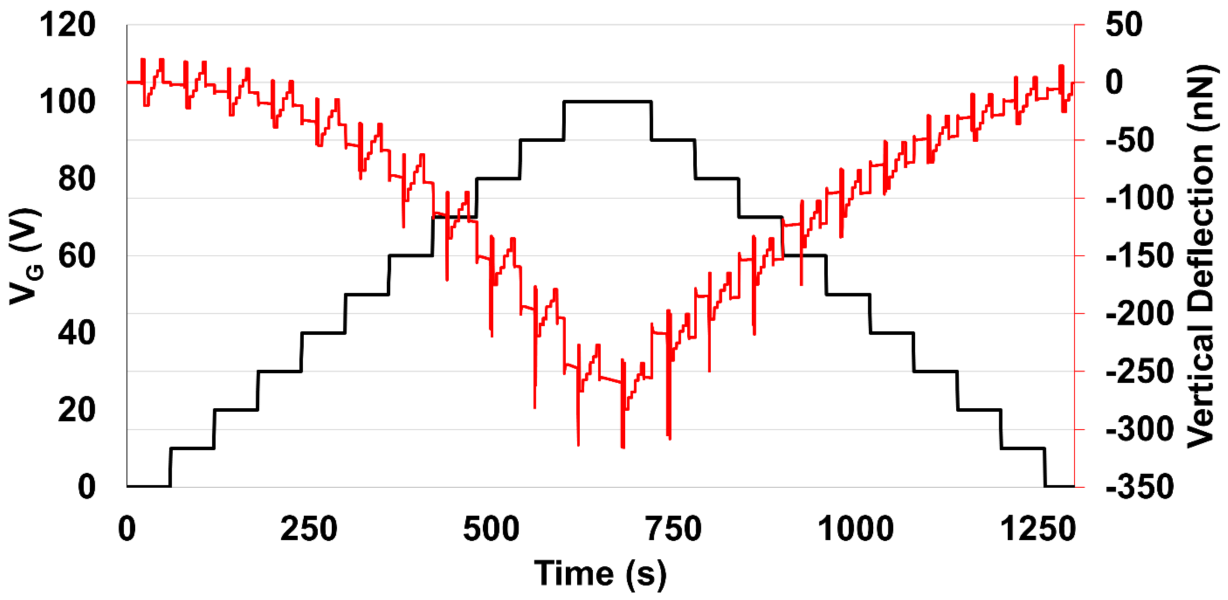

Supplementary Figure 13. Raw data for the normal deflection of the AFM tip over the duration of a friction measurement. The black line shows the applied backgate potential while the red line shows the vertical force experienced by the tip, calculated by multiplying the raw deflection in Volts by the sensitivity in nm/V and spring constant in N/m. The deflection towards the surface (a negative vertical deflection) from the baseline before the power source is turned on is considered a positive value for the electrostatic attraction F_{el}. At each voltage step the tip is initially deflected and then approximately stable; the tip is approached to the surface after ~15 s and various loads are applied (in this case 6) to measure friction as a function of load at constant back gate potential, which appears as additional deflections at each potential. At the end of the friction measurements the tip retracts from the surface to again be deflected by only the electrostatic attraction. The sample rate of the vertical deflection is 10 Hz.

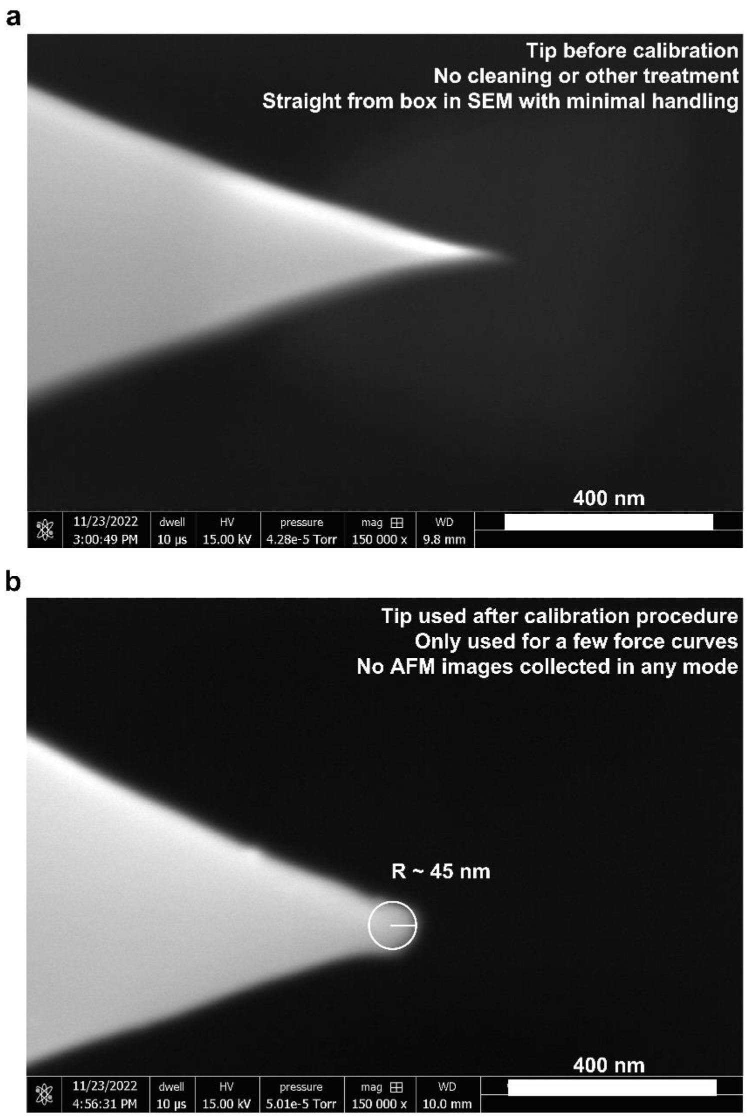

Supplementary Figure 14. **Quantification of initial tip breakage on first use of a silicon AFM tip in contact mode**. **a** SEM image of an unused AFM as provided by the manufacturer. **b** SEM image of the same tip after cleaning and contact mode calibration but before use in any further measurement. The tip radius has increased to ∼ 45 nm.

Supplementary References

1 Park, J. Y., Qi, Y., Ogletree, D. F., Thiel, P. A. & Salmeron, M. Influence of carrier density on the friction properties of siliconpnjunctions. *Physical Review B* **76**, 064108 (2007).

2 Wang, W., Dietzel, D. & Schirmeisen, A. Single-asperity sliding friction across the superconducting phase transition. *Sci Adv* **6**, eaay0165 (2020).

3 Calizo, I., Balandin, A., Bao, W., Miao, F. & Lau, C. Temperature dependence of the Raman spectra of graphene and graphene multilayers. *Nano Lett.* **7**, 2645 (2007).

4 Yang, Y., Brenner, K. & Murali, R. The influence of atmosphere on electrical transport in graphene. *Carbon* **50**, 1727 (2012).

5 Sang, Y., Dubé, M. & Grant, M. Thermal effects on atomic friction. *Physical Review Letters* **87**, 174301 (2001).

6 Yin, Y., Cheng, Z., Wang, L., Jin, K. & Wang, W. Graphene, a material for high temperature devices–intrinsic carrier density, carrier drift velocity and lattice energy. *Scientific reports* **4**, 1 (2014).

7 Massicotte, M., Soavi, G., Principi, A. & Tielrooij, K.-J. Hot carriers in graphene–fundamentals and applications. *Nanoscale* **13**, 8376 (2021).

8 Eyring, H. Viscosity, Plasticity, and Diffusion as Examples of Absolute Reaction Rates. *J. Chem. Phys.* **4**, 283 (1936).
